# Trueness of ten intraoral scanners in determining the positions of simulated implant scan bodies

**DOI:** 10.1038/s41598-021-82218-z

**Published:** 2021-01-28

**Authors:** Ryan Jin Young Kim, Goran I. Benic, Ji-Man Park

**Affiliations:** 1 Department of Dental Science and Dental Research Institute, School of Dentistry, Seoul National University, Seoul, 03080 South Korea; 2 Clinic of Fixed and Removable Prosthodontics and Dental Material Science, Center for Dental Medicine, University of Zurich, 8032 Zurich, Switzerland; 3 Department of Prosthodontics, College of Dentistry, Yonsei University, Seoul, 03722 South Korea

**Keywords:** Dental equipment, Dental implants, Fixed prosthodontics, Fixed prosthodontics

## Abstract

Few investigations have evaluated the 3-dimensional (3D) accuracy of digital implant scans. The aim of this study was to evaluate the performance of 10 intraoral scanners (IOSs) (CEREC Omnicam, CEREC Primescan, CS 3600, DWIO, i500, iTero Element, PlanScan, Trios 2, Trios 3, and True Definition) in obtaining the accurate positions of 6 cylinders simulating implant scan bodies. Digital scans of each IOS were compared with the reference dataset obtained by means of a coordinate measuring machine. Deviation from the actual positions of the 6 cylinders along the XYZ axes and the overall 3D deviation of the digital scan were calculated. The type of IOSs and position of simulated cylindrical scan bodies affected the magnitude and direction of deviations on trueness. The lowest amount of deviation was found at the cylinder next to the reference origin, while the highest deviation was evident at the contralateral side for all IOSs (*p* < 0.001). Among the tested IOSs, the CEREC Primescan and Trios 3 had the highest trueness followed by i500, Trios 2, and iTero Element, albeit not statistically significant (*p* > 0.05), and the DWIO and PlasScan had the lowest trueness in partially edentulous mandible digital implant scans (*p* < 0.001).

## Introduction

An intraoral scanner (IOS) is a device used to translate 3-dimensional (3D) geometric information of intraoral structures into digital data. With the improvements in accuracy and convenience together with the affordable cost of IOSs since its first appearance more than 30 years ago, use of IOSs in daily dental practice is increasing^1–3^.

Performance of traditional impressions involve placing a material-loaded tray into the patient’s mouth, allowing it to set around the patient teeth for a certain period of time, and then removing it^1^. However, traditional fabrication of dental casts could be simplified using an IOS, which creates a digital 3D dental model from which dental restorations could be fabricated. When a physical dental cast is required, subtractive (milling) or additive technologies could be employed to construct a positive model from the digital dataset.

Dental restorations such as crowns, bridges, inlays, and veneers should precisely fit onto prepared teeth to enhance their longevity by minimizing complications associated with misfits between tooth substrate and restoration^4^. This fundamental principle also applies to components of dental implant prostheses and restorations^5^. Therefore, discrepancies between implant components should be minimal. In digital implant rehabilitation, the accuracy of IOSs relates to the final outcome of implant treatment because implant abutments and prostheses are constructed from digital models obtained by IOSs^6–8^. Since the size of the scanning window of IOSs is limited to allow manipulation of the scanner wand within the confined space of the oral cavity, a series of scan images is captured to create a virtual 3D model. During data reconstruction, dimensional changes are inevitable when images are stitched together at overlapping areas^9^. This discrepancy would translate into more clinical time required for adjustment of a restoration or prosthesis.

According to the definition provided by ISO 5625-1, trueness refers to the level of agreement between the arithmetical mean of a large number of tests and the true or accepted value^10^. The superimposition technique has been employed where the digital dataset of IOSs is overlaid onto the corresponding reference dataset to evaluate the trueness of IOSs by analyzing their relative deviation from the reference spatial information^11^. Since the accurate digital dataset of a reference model is a crucial prerequisite for subsequent reliable deviation measurements, a highly accurate industrial-level 3D model scanner should be used to minimize possible misleading information. Implementation of more accurate tools would be desirable for such micro-scale evaluations. Coordinate measuring machines (CMM) are regarded as the most accurate tool in dimensional metrology under the given conditions^12, 13^. However, information on the trueness of various IOSs for digital scan of implant scan bodies using CMM is limited.

Therefore, the purpose of this study was to evaluate the trueness of 10 IOSs for acquiring the accurate positions of simulated implant scan bodies on a partially edentulous model by analyzing the numerical spatial information of the master cast obtained from a CMM with the digital datasets of IOSs. The null hypotheses of this study were that there would be no significant difference in 3D trueness among the tested IOSs and that scan body position would have no effect on trueness.

## Methods

**Figure 1:**
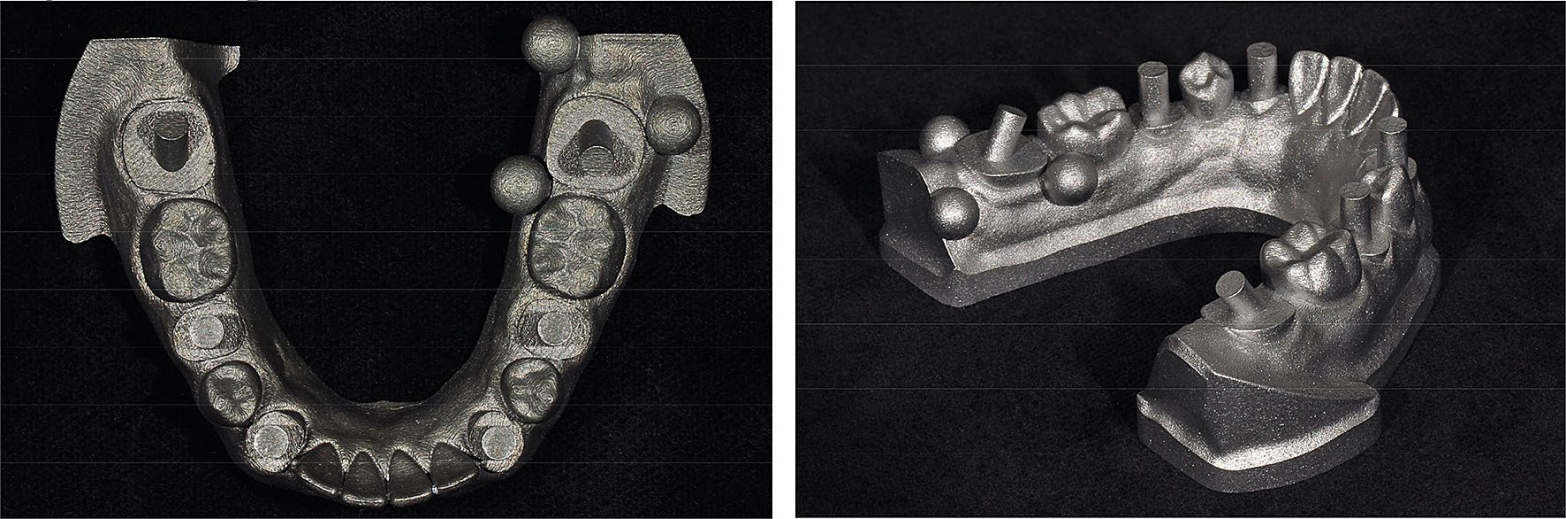
3D printed Co–Cr master model.

To fabricate a master model, a dentate complete-arch mandibular model (A50H-Set; Nissin Dental Products, Kyoto, Japan) was modified by cutting away the crown aspects of the bilateral second molars, second premolars, and canines. The modified dentiform was scanned with an industrial-grade scanner (StereoScan neo; AICON 3D Systems, Braunschweig, Germany) to obtain a digital mode, which was then imported to reverse engineering software (Rapidform 2004; Inus Technology, Seoul, Korea) to design 1) a cylinder (2 mm in diameter, 7 mm in height) at each of the 6 trimmed teeth and 2) three reference spheres with a diameter of 3.5 mm around the mandibular left second molar. The cylinders were added to simulate implant scan bodies. Except for the bilateral-most distal cylinders that were inclined 30 degrees, the other cylinders were positioned 90 degrees to the model. A 3D printer (Eosint M270; EOS, Krailling, Germany) was used to fabricate a Co-Cr master model using direct metal laser sintering technology (Fig. 1).

Digital scans of the master model were collected using 10 IOSs (CEREC Omnicam, CEREC Primescan, CS 3600, DWIO, i500, iTero Element, PlanScan, Trios 2, Trios 3, and True Definition) (Table 1). Scanning was performed by an operator at room temperature of 23 (± 2) °C in accordance with ISO 554^14^.

**Table 1:** Characteristics of intraoral scanners.

System	Manufacturer	Scanner Technology	Software Version	Light Source	Acquisition Method	Necessity of Coating
CEREC Omnicam	Dentsply Sirona	Active triangulation with strip light projection	4.6	Light	Video sequence	None
CEREC Primescan	Dentsply Sirona	Confocal microscopy	5	Light	Video sequence	None
CS 3600	Carestream Dental	Active triangulation (Stream projection)	1.0	Light	Video sequence	None
DWIO	Dental Wings	Active triangulation	1.0	LED light	Video sequence	None but occasionally
i500	MEDIT	Dual camera optical triangulation	1.1.1.2	Light	Video sequence	None
iTero Element	Align Technology	Parallel confocal microscopy	1.60	White LED light	Video sequence	None
PlanScan	Planmeca	Laser triangulation	5.7	Laser	Video sequence	None
Trios 2	3shape	Confocal microscopy	1.3.2.1	Light	Video sequence	None
Trios 3	3shape	Confocal microscopy	1.4.7.5	Light	Video sequence	None
True Definition	3M	Active wavefront sampling	4.2	Light	Video sequence	Yes

For all the IOSs, scanning was initiated on the occlusal surface from the left second molar to the contralateral end, followed by the lingual and buccal surfaces. Additional scans were performed to capture voided areas of the cylinders and spheres that were critical for measurement. The scanning procedure was performed 10 times for each IOS.

The CMM (Infinity 12.10.6; Leitz Messtechnik, Wetzlar, Germany) was used to measure the XYZ coordinates of each cylinder on the master model at a certified metrologic center (KITECH, Cheonan, Korea). According to ISO 10360-2^15^, the specific CMM has a length measuring error of 0.5 ± L/1000 μm and repeatability range of 0.2 μm. After measuring the master model for 10 times with the CMM, the averaged XYZ coordinates for each cylinder position represented the true reference value.

The reference origin point of the measurement was set to the center of the reference sphere located at the buccal aspect of the left second molar. The plane connecting the centers of the three reference spheres formed the XY plane. The Y-axis, denoting the anterior–posterior direction in the XY plane, was defined as a line perpendicular to a line formed from the origin to the midline connecting the centers of the two lingually located spheres. The X-axis, denoting the medial–lateral direction in the XY plane, was defined as a line perpendicular to the Y-axis. The Z-axis denotes the coronal-cervical direction from the origin perpendicular to the XY plane.

For subsequent evaluations, the XYZ coordinates were obtained at the centroid of the top surface of each of the 6 cylinders. The trueness values of the 10 IOSs at each cylinder position were calculated by subtracting the coordinate values from those of the reference CMM values in the XYZ axes. At each cylinder, the distance between two points, obtained by the CMM and IOS, in 3D Euclidean space was calculated to determine the 3D deviation of the at each cylinder using the following formula^16^ derived from the Pythagorean theorem:

$$3{\text{D}}\;{\text{deviation}}_{{\text{i}}} = \sqrt {\left( {{\text{r}}_{{{\text{ix}}}} - {\text{s}}_{{{\text{ix}}}} } \right)^{2} + \left( {{\text{r}}_{{{\text{iy}}}} - {\text{s}}_{{{\text{iy}}}} } \right)^{2} + \left( {{\text{r}}_{{{\text{iz}}}} - {\text{s}}_{{{\text{iz}}}} } \right)^{2} }$$

where r is the reference coordinate, s is the coordinate in each IOS, i is the cylinder position, and x, y, and z are the X, Y, and Z-axis, respectively.

For statistical evaluation, data were analyzed using SPSS 23.0 (IBM, Armonk, NY, USA). The Shapiro–Wilk test was carried out to verify the normality of each variable. The median trueness values of the IOSs were analyzed using the Kruskal–Wallis test, followed by Mann–Whitney U test and Bonferroni correction for pairwise comparisons at a significance level of 0.05.

## Results

**Figure 2:**
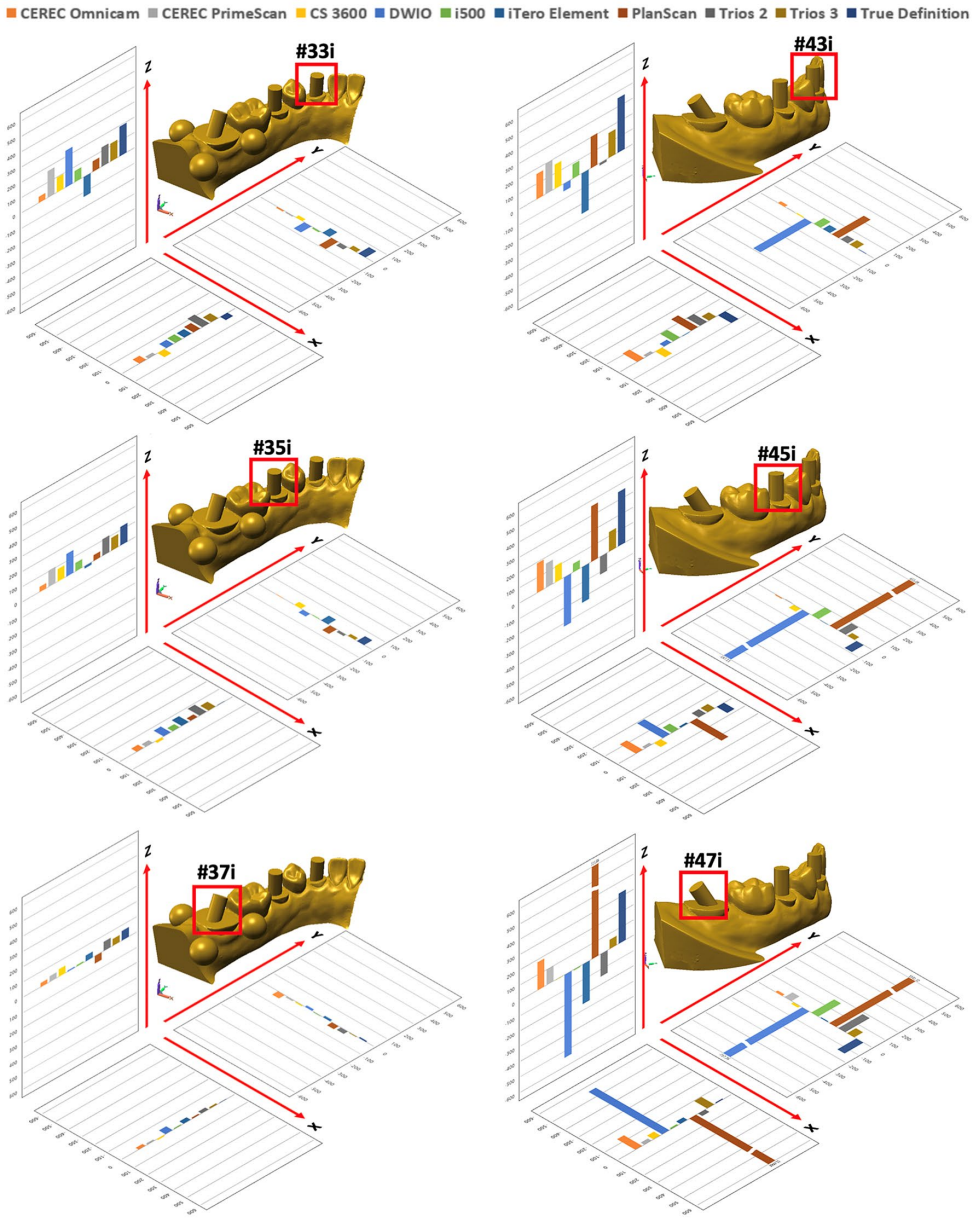
Trueness values (μm) of IOSs at each cylinder position in XYZ axes.

Trueness values in the XYZ axes for each scanner are shown in Table 2 and Fig. 2. Compared to the reference CMM points, each IOS exhibited variable directions and magnitudes of deviation depending on cylinder positiont. The deviation increase in the Z-axis was greater than that on the X- or Y-axis (*p* < 0.001). When the 3D deviations from all the cylinder positions were combined, the overall deviation was 138.98 μm in the Trios 3, 142.04 μm in the CEREC Primescan, 155.80 μm in the i500, 171.45 μm in the Trios 2, 192.37 μm in the iTero Element, 216.40 μm in the CEREC Omnicam, 254.30 μm in the CS 3600, 274.54 μm in the True Definition, 303.22 μm in the PlanScan, and 337.19 in the DWIO. The Trios 3 and CEREC Primescan exhibited the lowest deviation, albeit not statistically significant, compared with the i500, Trios 2, and iTero Element (*p* > 0.05), while the DWIO and PlanScan yielded significantly greater deviation among the tested IOSs (*p* < 0.001).

**Table 2:**
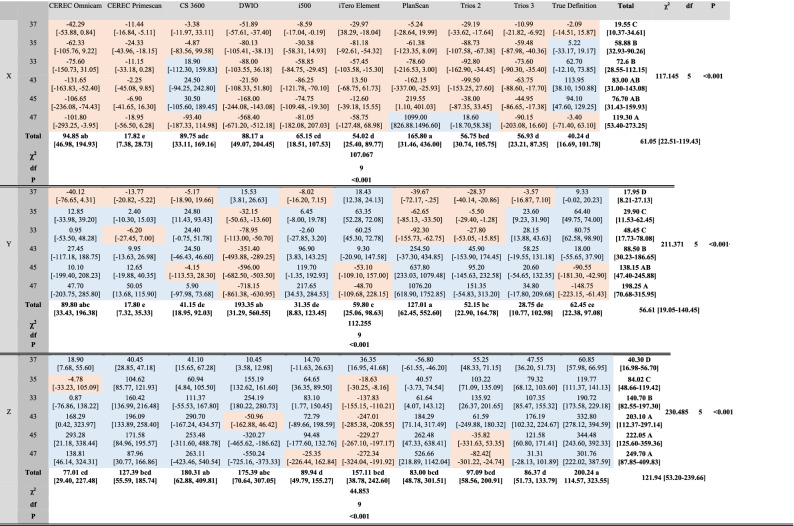
Trueness values (μm) of IOSs at each cylinder position in XYZ axes.

χ^2^, chi-square; df, degrees of freedom; *p*, *p*-value.

Interquartile ranges [1st quartile, 3rd quartile] are in parentheses.

Different uppercase letters within the same column indicate statistically significant differences among cylinder positions; different lowercase letters within the same row indicate statistically significant difference among IOSs (multiple comparison by Mann–Whitney U test with Bonferroni correction) (*p* < 0.05).

Positive (in blue shade) and negative values (in red shade) indicate deviation to the right and left in X-axis, forwards and backwards in Y-axis, upwards and downwards in Z-axis, respectively.

Absolute values were used for statistical analysis.

All the IOSs yielded greater deviation from the left second molar area, nearest to the reference origin, toward the right second molar area (*p* < 0.001) (Table 3). This deviation was more marked in the DWIO and PlanScan (*p* < 0.001).

**Table 3:** Overall 3D deviation (μm) at each cylinder position.

	CEREC Omnicam	CEREC Primescan	CS 3600	DWIO	i500	iTero Element	PlanScan	Trios 2	Trios 3	True Definition	χ^2^	df	*p*	Total	χ^2^	df	*p*
37	72.40 Cabc [53.17, 95.30]	43.35 Ccd [39.52, 50.52]	48.69 Cbcde [35.17, 75.91]	53.39 Dabcd [48.64, 60.04]	28.11 Dd [21.32, 32.09]	50.35 Dabcd [47.79, 58.48]	77.13 Da [69.11, 114.13]	71.50 Bab [63.89, 82.68]	51.52 Bbcd [44.44, 52.74]	64.71 Cabc [60.14, 68.65]	**60.124**	**9**	** < 0.001**	**54.14 D** **[46.32, 68.97]**	**365.112**	**5**	** < 0.001**
35	98.10 BCabc [78.68, 201.88]	113.76 BCabc [102.05, 123.80]	129.08 BCabc [96.33, 117.05]	178.53 CDa [149.46, 189.94]	83.06 CDc [72.07, 99.24]	102.21 CDc [86.11, 116.66]	134.75 CDabc [97.66, 194.82]	133.95 ABab [120.01, 163.26]	111.37 ABabc [101.64, 125.59]	141.69 BCab [126.83, 154.54]	**36.032**		** < 0.001**	**119.54 C** **[96.42, 153.70]**			
33	129.77 ABCabc [115.71, 287.20]	167.49 ABabc [150.39, 218.14]	180.18 ABCabc [136.10, 297.07]	282.65 BCDa [206.81, 315.32]	124.85 BCDa [98.30, 155.24]	167.44 BCDabc [135.16, 192.86]	248.40 BCDabc [146.67, 305.86]	203.00 Aabc [147.18, 237.29]	143.79 Abc [115.96, 163.13]	219.56 ABCabc [197.64, 256.79]	**28.867**		** < 0.001**	**181.59 B** **[132.31, 248.75]**			
43	318.27 ABabc [182.06, 533.90]	204.40 Ac [168.80, 259.95]	425.65 ABabc [250.22, 713.94]	414.13 ABCa [360.23, 509.14]	220.68 ABabc [166.15, 279.1]	287.17 ABabc [216.59, 364.27]	444.76 ABCa [298.39, 631.86]	249.70 Aabc [179.34, 432.48]	226.23 Abc [168.55, 269.60]	390.24 Aabc [328.82, 423.11]	**34.451**		** < 0.001**	**292.34 A** **[206.81, 417.46]**			
45	343.07 Aabcdef [237.33, 550.84]	179.66 ABf [140.40, 205.74]	463.06 Aabcde [273.07, 893.66]	751.36 ABa [626.11, 818.77]	267.78 ABdef [168.75, 322.48]	279.92 ABcdef [226.74, 304.33]	793.99 ABa [525.76, 1369.75]	236.95 Abcdef [164.00, 473.00]	203.74 Aef [135.34, 250.42]	402.70 Aabcde [355.84, 420.82]	**57.749**		** < 0.001**	**310.90 A** **[201.49, 562.87]**			
47	372.48 Aabcde [269.31, 617.73]	148.54 ABe [59.71, 200.67]	460.14 Aabcd [342.97, 961.39]	1125.80 Aa [983.12, 1253.36]	371.76 Aabcde [272.47, 572.92]	327.44 Abcde [291.41, 387.01]	1616.21 Aa [1189.91, 2742.02]	260.97 Acde [132.64, 541.68]	220.78 Ade [175.22, 283.18]	394.84 Aabcde [348.08, 437.00]	**66.067**		** < 0.001**	**373.61 A** **[240.87, 770.01]**			
**χ**^**2**^	**34.957**	**35.273**	**37.704**	**56.425**	**49.798**	**49.41**	**48.697**	**32.167**	**32.751**	**49.378**							
**df**						**5**											
**P**	** < 0.001**	** < 0.001**	** < 0.001**	** < 0.001**	** < 0.001**	** < 0.001**	** < 0.001**	** < 0.001**	** < 0.001**	** < 0.001**							
**Total**	**216.40 abcd** **[98.18, 347.67]**	**142.04 e** **[89.22, 198.12]**	**254.30 abc [105.96, 499.12]**	**337.19 a** **[178.07, 760.16]**	**155.80 cde** **[80.95, 274.85]**	**192.37 bcde** **[90.71, 302.86]**	**303.22 a** **[126.09, 933.56]**	**171.45 bcde** **[119.26, 264.22]**	**138.98 de** **[82.42, 212.00]**	**274.54 abc [141.32, 400.78]**	**194.05 [109.09, 339.51]**						
**χ**^**2**^						**70.951**											
**df**						**9**											
**P**						** < 0.001**											

χ^2^, chi-square; df, degrees of freedom; *p*, *p*-value.

Interquartile ranges [1st quartile, 3rd quartile] are in parentheses.

Different uppercase letters within the same column indicate statistically significant differences among cylinder positions; different lowercase letters within the same row indicate statistically significant difference among IOSs (multiple comparison by Mann–Whitney U test with Bonferroni correction) (*p* < 0.05).

**Figure 3:**
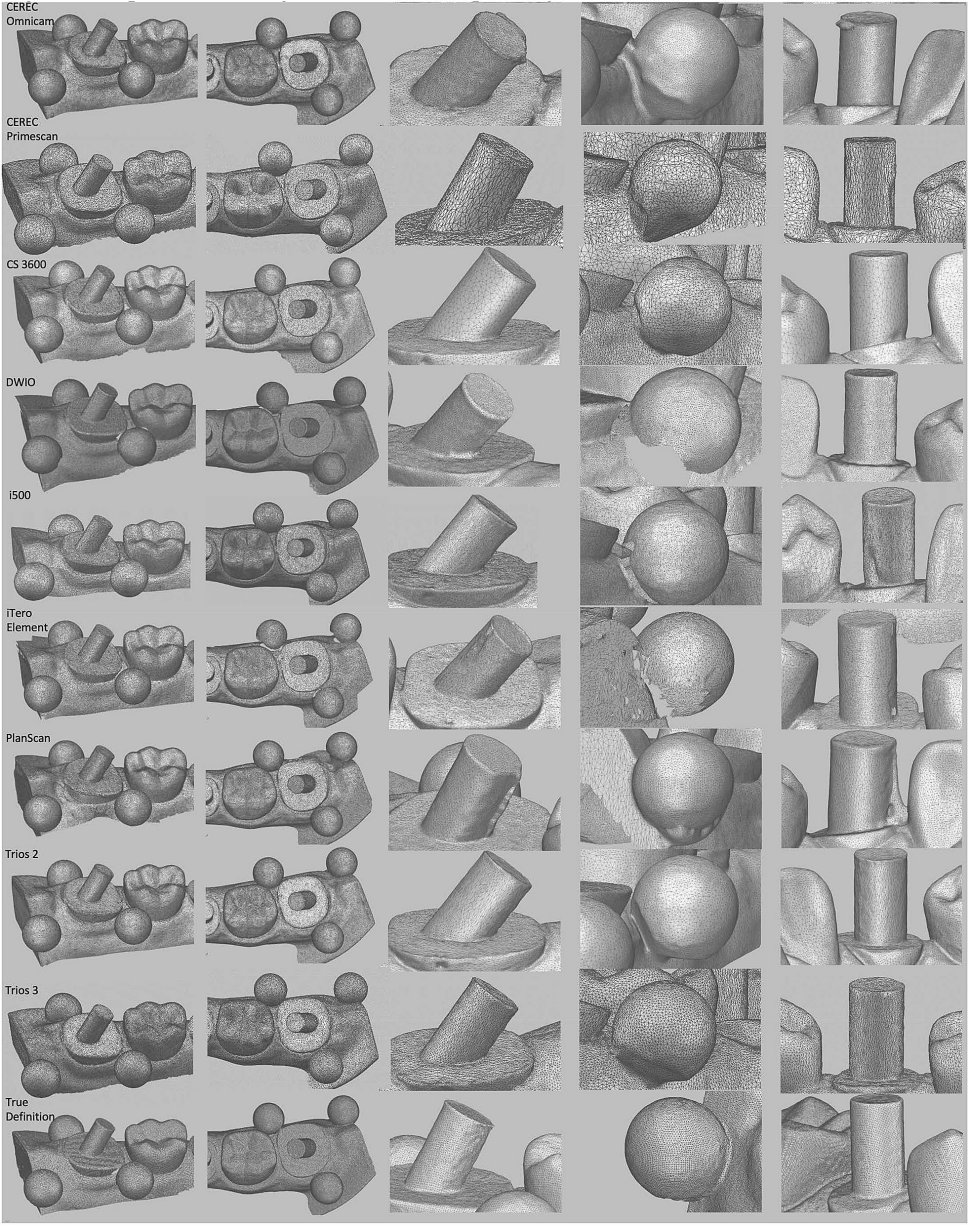
Representative digital models corresponding to master model.

Figure 3 shows the representative images of the digital models, some with frequently observed surface features.

## Discussion

In the present study trueness evaluation of IOSs relative to the reference CMM demonstrated differences in deviation magnitude depending on type of IOS. The lowest 3D deviations were obtained in the Trios 3 and CEREC Primescan, followed by the i500, Trios 2, iTero Element, CEREC Omnicam, CS 3600, and True Definition, while the DWIO and PlanScan yielded greater deviation among the tested IOSs. Therefore, the findings of this study do not support the first hypothesis that tested IOSs have similar 3D trueness. Digital scans deviated with distance from the reference origin; the further was the scan from the origin, the greater was the deviation. This result is in line with previous studies confirming the diminished trueness of digital implant scans due to accumulation of errors during image stitching^6, 9, 17–21^, rejecting the second hypothesis that scan body position would have no effect on trueness.

With regard to trueness in the XYZ directions, there was a tendency for the Z-axis to be the most error-prone direction. This discrepancy seems to be associated with difficulty in maintaining a steady distance between the IOS and scanning object when the IOS is manipulated in the oral cavity while watching a monitor screen. The difference in visual-motor integration causes inconsistent distance control which in turn may lead to scanned images that are not ideally focused, thus triggering errors in the process of overlapping images. This phenomenon seems to occur more frequently when scanning the occlusal surface, leading to greater deviation in the Z-axis when compared to XY-axes.

Notably, all IOSs showed an increase in trueness values between the cylinders positioned at the canine regions, implying that scanning errors occur more readily in the anterior region. In this regard, Ender et al.^22^ analyzed the accuracy of digital scans at three regions of interest of complete-arch, posterior segment, and anterior segment; and found higher deviations within the anterior segment, resulting in lower accuracy for the complete-arch. The authors attributed the greater deviation in the anterior region to the relatively limited morphological features compared with the posterior segment. In this study, the DWIO and PlanScan were the only groups that exhibited significant deviations along the cylinder positions on the contralateral side of the reference origin, indicating that the performance of the 2 IOSs was not as accurate as the other IOSs for complete-arch scanning.

Recently, the accuracy levels of 8 IOSs in digital implant scans were evaluated by Di Fiore et al.^23^, who demonstrated a similar order of trueness of IOSs, even though the numerical values were different, to those from the present study. According to previous studies, the most accurate IOSs in digital scans for complete-arches were the i500 and Trios^6^, CS 3600 and Trios^21^, Trios^23^, True Definition and Trios^24^, and CS 3600^25^. The differences in the outcomes found between the studies might be explained through variations in methodologies, including type of master model, method for determining trueness, and number of IOSs. Osnes et al.^26^ evaluated precisions of 6 IOS for scanning a complete edentulous maxillary arch. Based on the previous study, they casted doubt on the trueness of the Planmeca Emerald and DWIO, both of which showed the greatest mean errors compared to the Aadva, CEREC Omnicam, Trios 3, and True Definition^26^. In line with previous studies^22, 27^, the recently released CEREC Primescan was more accurate in comparison to the previous CEREC Omnicam system. This might be explained by a larger scanning window with a greater field of view in the CEREC Primescan, enabling faster scanning and potentially reducing the risk of errors from matching overlapping data. In addition, differences in processing software may account for the performance difference between the two IOSs^22, 28^. With regards to the two Trios systems, the Trios 3 performed better than the Trios 2, albeit not statistically significant. However, their combined effect was not as significant as that of the CEREC system.

The qualitative aspects of the IOSs were different when the digital models were compared. Polygon meshes created by each IOS system was not identical in terms of number and geometry. This is reflected in the typical features such as edge sharpness and surface smoothness. The final digital model is reconstructed by specific algorithms designed to address redundancies, noise, and incompatible or missing data. The combined effect of different scanning technology for image acquisition and data processing software used in each IOS system are responsible for varying polygon mesh features and trueness at different scan sites^9^.

The CMM has been used to set a reference to which the accuracy of digital scans was compared to the conventional impression techniques^29–33^. Based on the results of previous studies, the optimal method could not be proven because only one IOS system was used for digital scans in each study. For the entire surface evaluation, 3D industrial-grade scanners have been widely used to obtain reference data to which datasets of a digital model are compared by the superimposition technique using 3D analyzing software. Thus, the general pattern of deviation could be readily inspected over the entire region of interest. However, 3D reference scanner was not used in this study, even with its proven accuracy. Instead, reference datasets were produced by a CMM since it is a very precise measurement device that has been regarded as the gold standard in metrology for quality control of a product^12, 13^. Therefore, CMM was an ideal tool for this study because specific points of the digital model, the centroid of the top surface of each cylinder, were directly compared for 3D XYZ deviation and not at a region of broad-area surfaces. Unlike 3D scanners, as the XYZ coordinates for specific points are measured when a stylus on a probe contacts the surface, some of the limiting factors of traditional CMM include slow acquisition speed in cases of multiple area measurements and difficulty capturing fine geometrical features^11^.

The design of implant scan bodies is highly variable^34^. Although the accuracy of implant positioning could be influenced by the different design features of scan bodies^16, 35^, a simplified cylindrical-shaped scan body design was used in the present study because it is the most commonly available design that could be readily reverse engineered to simulate scan bodies. This allowed us to focus on the 3D evaluation of digital implant scan, while at the same time eliminating variables associated with the design of the scan body.

The limitations of the present study include collection of digital impressions in an in vitro model, which may differ from real clinical scenarios where the outcome could be influenced by factors such as patient movement and presence of soft tissue and moisture. With regard to scanning path, the identical scanning strategy was used for all IOSs even though scanning strategies appear to show a difference in accuracy depending on IOS^22, 23, 36, 37^. Furthermore, when compared to modified dentate master model digitized in this study, digital impression of an edentulous model may result in different performance of the IOSs due to the difference in anatomical landmarks. Since the result of a trueness accuracy analysis depends on the methodology, despite the demonstration of the trueness of 10 IOSs at various sites during digital implant impression procedures in this study, the results from this study needs to be interpreted with caution.

In conclusion, the IOSs exhibited variable magnitudes and directions of deviation at each position of the simulated cylindrical scan bodies. The deviation increased at cylinders positioned further from the reference origin. Overall, the CEREC Primescan and Trios 3 had the highest trueness in partially edentulous mandible digital implant scans, followed by the i500, Trios 2, and iTero Element, albeit not statistically significant (*p* > 0.05). However, the DWIO and PlanScan exhibited significantly more distortion among the IOSs, particularly in the contralateral side of the reference origin (*p* < 0.001). It would be recommended to use an IOS that is capable of producing accurate complete-arch scan, especially for fabrication of long-span prostheses or appliances.
